# Impact of sandwiched strain periodic multilayer AlN/GaN on strain and crystalline quality of *a*-plane GaN

**DOI:** 10.1038/s41598-021-89201-8

**Published:** 2021-05-06

**Authors:** Anas Kamarundzaman, Ahmad Shuhaimi Abu Bakar, Adreen Azman, Al-Zuhairi Omar, Noor Azrina Talik, Azzuliani Supangat, Wan Haliza Abd Majid

**Affiliations:** 1 Low Dimensional Materials Research Centre, Department of Physics, Faculty of Science, University of Malaya, 50603 Kuala Lumpur, Malaysia; 2 Nanotechnology Research Centre, Department of Physics, Faculty of Science and Mathematics, Universiti Pendidikan Sultan Idris, 35900 Tanjong Malim, Perak Malaysia

**Keywords:** Electronics, photonics and device physics, Photonic devices, Lasers, LEDs and light sources

## Abstract

We demonstrated high-quality single crystalline *a*-plane undoped-gallium nitride grown on a nonpatterned *r*-plane sapphire substrate via metal–organic chemical vapor deposition. The effect of four different numbers of sandwiched strain-periodic AlN/GaN multilayers on the strain state, crystal quality, optical and electrical properties was investigated. Field emission scanning electron microscopy and atomic force microscopy showed that the surface morphology was improved upon insertion of 120 pairs of AlN/GaN thin layers with a root-mean-square roughness of 2.15 nm. On-axis X-ray ω-scan rocking curves showed enhanced crystalline quality: the full width at half maximum decreased from 1224 to 756 arcsec along the [0001] direction and from 2628 to 1360 arcsec along the [1–100] direction for *a*-GaN grown with 120 pairs of AlN/GaN compared to *a*-GaN without AlN/GaN pairs. Reciprocal space mapping showed that *a*-plane GaN with a high number of AlN/GaN pairs exhibits near-relaxation strain states. Room-temperature photoluminescence spectra showed that the sample with the highest number of AlN/GaN pairs exhibited the lowest-intensity yellow and blue luminescence bands, indicating a reduction in defects and dislocations. The *a*-plane InGaN/GaN LEDs with 120 pairs of SSPM-L AlN/GaN exhibited a significant increase (~ 250%) in light output power compared to that of LEDs without SSPM-L AlN/GaN pairs.

## Introduction

The performance of gallium nitride (GaN)-based light-emitting devices has improved tremendously in recent years. Outstanding performance has recently been reported for InGaN/GaN-based LEDs along *c*-plane orientation^1,2^. However, it is commonly known that growing LEDs along the *c*-plane results in high built-in spontaneous and piezoelectric polarization, which leads to the quantum confined Starks effect (QCSE)^3–6^. Therefore, several groups have expended considerable effort in growing epitaxial layers along nonpolar surfaces in the absence of piezoelectric and polarization fields. The energy band of *a*-plane GaN lies perpendicular to the polarization field, which presents a significant advantage over *a*-plane devices with no polarization in the active region^7,8^. The resulting increment in the electron–hole recombination rate increases the quantum efficiencies. Hence, this growth strategy produces considerable advantages for light emitting devices.

However, the growth of *a*-plane GaN on foreign substrates has been hindered thus far by the formation of high densities of basal stacking faults (BSFs) and threading dislocations (TDs) during the growth process^9,10^. The anisotropic properties of nonpolar GaN have become the main challenge in growing *a*-plane GaN. The lattice mismatch and thermal expansion coefficient differential along the [11–20] and [0001] directions induce distortions in the grown GaN unit cell that interrupt hexagonal symmetry. This phenomenon promotes defects and increases the densities of dislocations, such as BSFs and TDs, leading to challenges in determining the lattice parameters, because more variables need to be considered^11–14^. Several strategies have recently been developed to overcome this challenge, including patterning of hole arrays, patterning of stripped SiO_2_ and regrowth on Ni nanopatterned *a*-GaN templates^15–17^. However, these techniques are time-consuming because other processes (ex situ) are required to supplement the epitaxial process.

Growing GaN along the *a*-plane direction limits the accessible lattice points for reciprocal space mapping using X-ray diffraction (XRD) measurements; thus, different approaches have been developed to estimate the lattice parameters of *a*-plane GaN. Darakchieva et al*.* suggested measuring 2θ-ω symmetrically at 90° intervals of the azimuth angle and at a few asymmetric planes within the edge-symmetric geometry^18^. Roder et al*.* followed a different approach by measuring the full width at half maximum (FWHM) from the ω-scan at nine different planes under symmetric, asymmetric and skew symmetric conditions within the diffraction geometry^19^. In this study, we used reciprocal space mapping (RSM) to directly access the (11–22) lattice point to measure the lattice size along the [11–20] and [0001] directions, while considering other important parameters of the distorted wurtzite structure.

Considering the abovementioned factors, we inserted a sandwiched strain-periodic multilayer (SSPM-L) structure into our *a*-plane epitaxial layer, that is, an AlN/GaN thin layer was sandwiched between two thick *a*-plane GaN layers. The objective of this study was to investigate how different numbers of SSPM-Ls enhance the surface morphology, crystalline quality and strain state of *a*-GaN epitaxial layers grown on *r*-sapphire for application to light-emitting devices. The effect of different numbers of SSPM-L AlN/GaN pairs on the electrical properties of InGaN/GaN-based LEDs grown on our *a*-GaN film was also investigated. The results showed the surface morphology, crystalline quality and strain state of the *a*-GaN epitaxial layer were clearly enhanced for a large number of SSPM-L AlN/GaN. The *a-*plane InGaN/GaN LEDs exhibited a high output power and high indium (In) incorporation for a InGaN/GaN multiple-quantum well (MQW) grown on a large number of SSPM-L AlN/GaN pairs.

## Experimental methods

**Figure 1:**
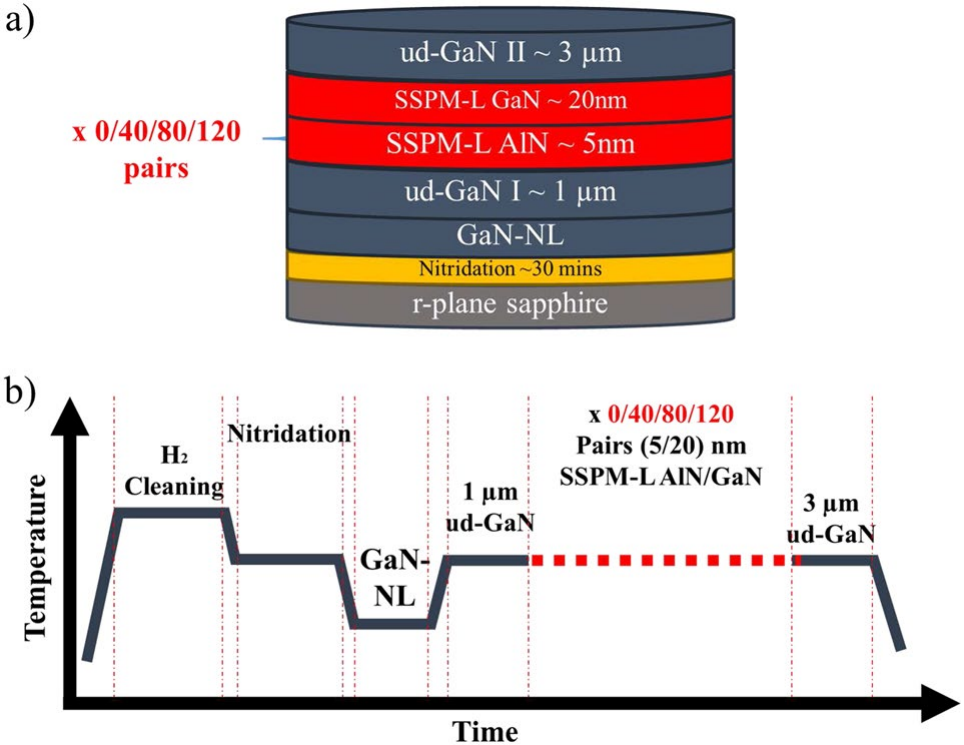
(**a**) Schematic and (**b**) epitaxial process flow for *a*-GaN film grown with and without SSPM-L AlN/GaN.

An *a*-plane GaN epitaxial layer (11–20) was grown on a two-inch nonpatterned (1–102) *r*-plane sapphire substrate via metal organic chemical vapor deposition (MOCVD) using a Taiyo Nippon Sanso SR2000 reactor with a horizontal flow. A total of four *a*-GaN films were grown using different numbers of SSPM-L AlN/GaN pairs, as shown in Fig. 1a, namely, 0, 40, 80 and 120 pairs, where the corresponding samples are denoted by S1, S2, S3 and S4, respectively. Trimethylgallium (TMGa), trimethylaluminium (TMAl), trimethylindium (TMI), biscyclopentadienylmagnesium (Cp_2_Mg), disilane (Si_2_H_6_) and ammonia (NH_3_) precursors were used as Ga, Al, In, Mg, Si and N sources, respectively. Hydrogen (H_2_) was used as the main carrier gas for the epitaxial processes, and nitrogen (N_2_) was used as the carrier gas for the growth of InGaN/GaN MQW. The *r*-sapphire substrate was prepared for the epitaxial process by baking at 1125 °C in ambient H_2_ to remove surface contamination^20^, followed by a 10-min nitradation step at 1050 °C. Thereafter, a 90-nm thin GaN nucleation layer (NL) was grown at 700 °C as a nucleation site for the subsequent layer. The temperature was then increased to 1050 °C, and 20 SLM of TMGa flow was released into the reactor to grow 1-µm-thick GaN. Epitaxial growth continued as SSPM-L AlN/GaN was deposited with a thickness of 5/20 nm and different numbers of pairs (0, 40, 80 and 120). Subsequently, 3-µm-thick GaN was grown on SSPM-L AlN/GaN under the same conditions as the initial 1-µm-thick GaN was grown. The process flow for the epitaxial process is presented in Fig. 1b. The *a*-GaN epitaxial layer grown with different pairs of SSPM-L AlN/GaN was characterized using an Olympus optical microscope (OM), a Hitachi SU8220 field emission scanning electron microscope (FESEM), a Bruker Multimode 8 atomic force microscope (AFM), a Rigaku SmartLab high resolution X-ray diffractometer (HR-XRD) and a LabRAM HR Horiba (to perform room-temperature photoluminescence (RT-PL)). Subsequently, 500-nm-thick Si-doped GaN was grown on the *a*-GaN templates, followed by 5 pairs of InGaN/GaN MQW to serve as the active region. Finally, 100-nm-thick Mg-doped GaN was grown on the MQW. The IV characteristics of the LED were measured at the wafer stage using an Agilent Technologies B1505A power device analyzer/curve tracer. The wavelength emission was measured using an OceanView spectrometer.

## Results and discussions

**Figure 2:**
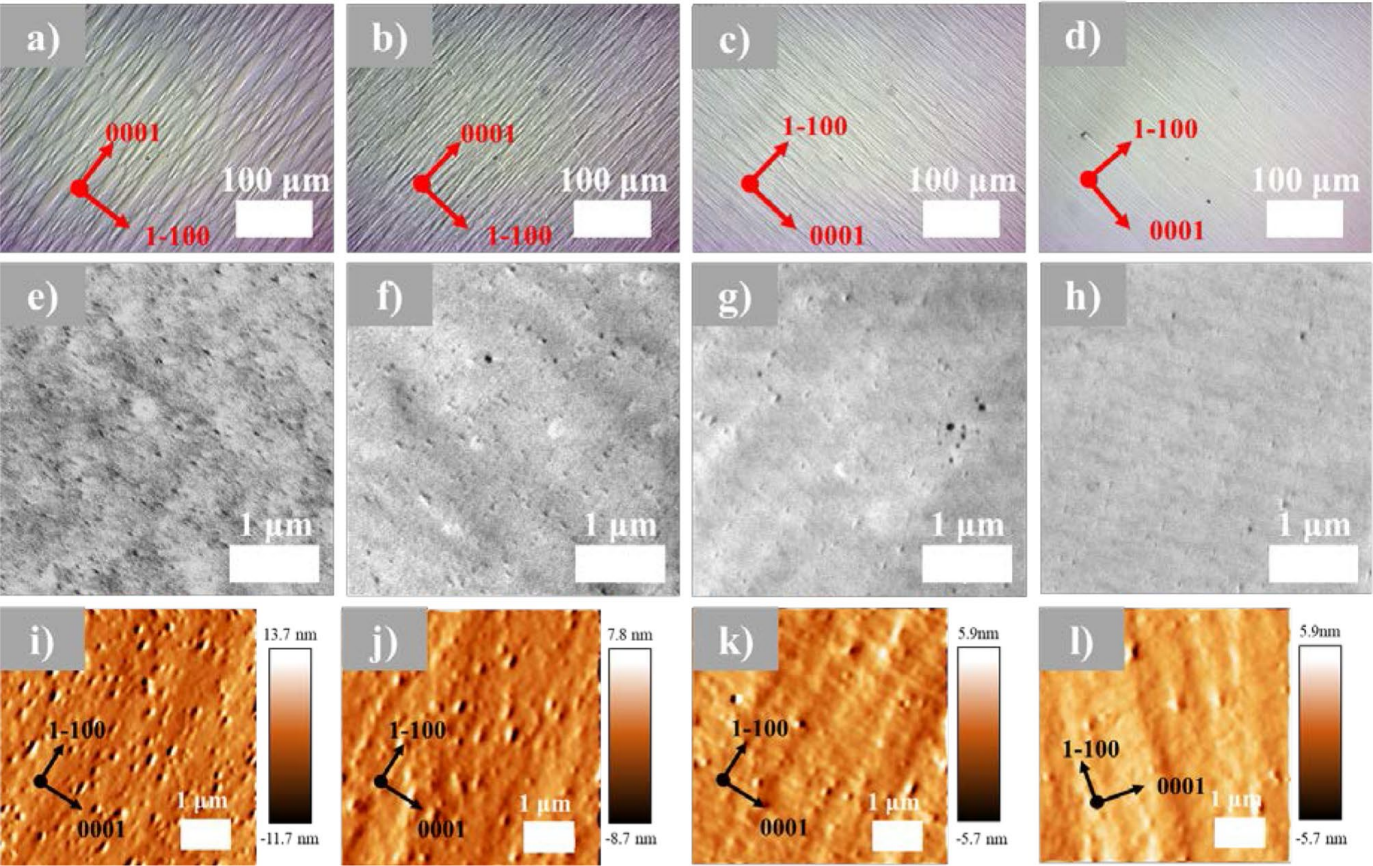
Surface structure images for samples S1, S2, S3 and S4: optical microscope images at 20 times magnification: (**a**–**d**), respectively; FESEM surface images: (**e**–**h**), respectively; and AFM images: (**i**–**l**), respectively.

Optical microscopy (OM) was first used to investigate the effect of SSPM-L AlN/GaN on the surface morphology. Figure 2a–d shows OM images for *a-*GaN grown with 0, 40, 80 and 120 pairs of SSPM-L AlN/GaN, respectively. All samples clearly exhibit anisotropy-induced stripe-like and arrowhead mosaic structures along (0001) c-plane direction, which are commonly observed for *a-*GaN growth^21–24^. Note that the stripe-like and arrowhead structures decreased in size as the number of SSPM-L AlN/GaN increased. As shown in Fig. 2a, sample S1 exhibited the highest surface roughness with a pronounced stripe-like structure and arrowhead, whereas sample S4 had a smoother film surface than the other samples. It is noteworthy that the stripe-like and arrowhead structures were generated from asymmetric lattice mismatches of 16.1% and 1.1% between the substrate and *a-*GaN film along the [1–100] and [0002] directions, respectively^25^. This result could be attributed to the different probabilities for surface atom incorporation and diffusion lengths along these non-identical crystallographic directions^26^.

Next, FESEM surface analysis was conducted to further investigate how the SSPM-L changed the surface morphology, and the results are shown in Fig. 2e–h. All the samples clearly exhibit black spots (voids), which could be attributed to v-pit defects in the *a*-GaN structure. This defect structure may have been caused by different growth rates along the [1–100] and [1000] directions and adatom diffusion kinetics during the growth process^27–29^. Moreover, the large asymmetric lattice mismatch between *a-*GaN and the substrate produces an in-plane strain distribution because of the absence of six-fold symmetry in the crystal arrangement^19^. Consequently, it is difficult for *a*-GaN to form a fully abrupt structure over the entire two-inch sapphire substrate. However, the sample with 120 pairs of SSPM-L AlN/GaN exhibits fewer voids than the other samples, assuming that the lowest number of defects were generated on the surface of this sample. Increasing the number of SSPM-L AlN/GaN pairs induced a suitable lattice size and improved the strain state for subsequent thick *a*-GaN growth. AFM measurements were carried out to further investigate the morphological behaviour of *a-*GaN films grown on different numbers of SSPM-L AlN/GaN pairs, as shown in Fig. 2i–l. The measured root-mean-square (RMS) roughness for samples S1, S2, S3 and S4 are 6.93 nm, 4.13 nm, 2.70 nm and 2.15 nm, respectively. These results suggest that the number of SSPM-L AlN/GaN pairs strongly affects the morphological structure of the grown *a-*GaN: *a-*GaN grown with 120 pairs of SSPM-L AlN/GaN had a ~ 68% lower surface roughness than *a-*GaN without SSPM-L AlN/GaN. All the AFM images show significant colouration similar to the FESEM images, and the v-pit defects observed in both the AFM and FESEM images contribute to an increase in the surface roughness of the grown GaN.

**Figure 3:**
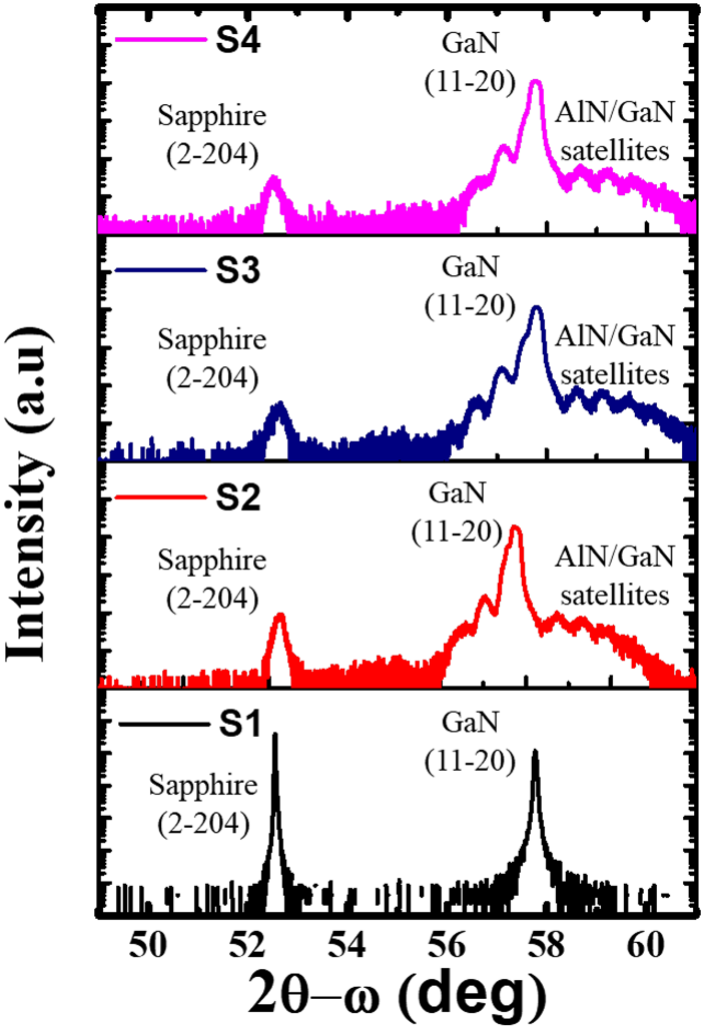
HR-XRD phase analysis 2θ-ω scan of *a*-GaN grown using 0, 40, 80 and 120 pairs of SSPM-L AlN/GaN.

**Figure 4:**
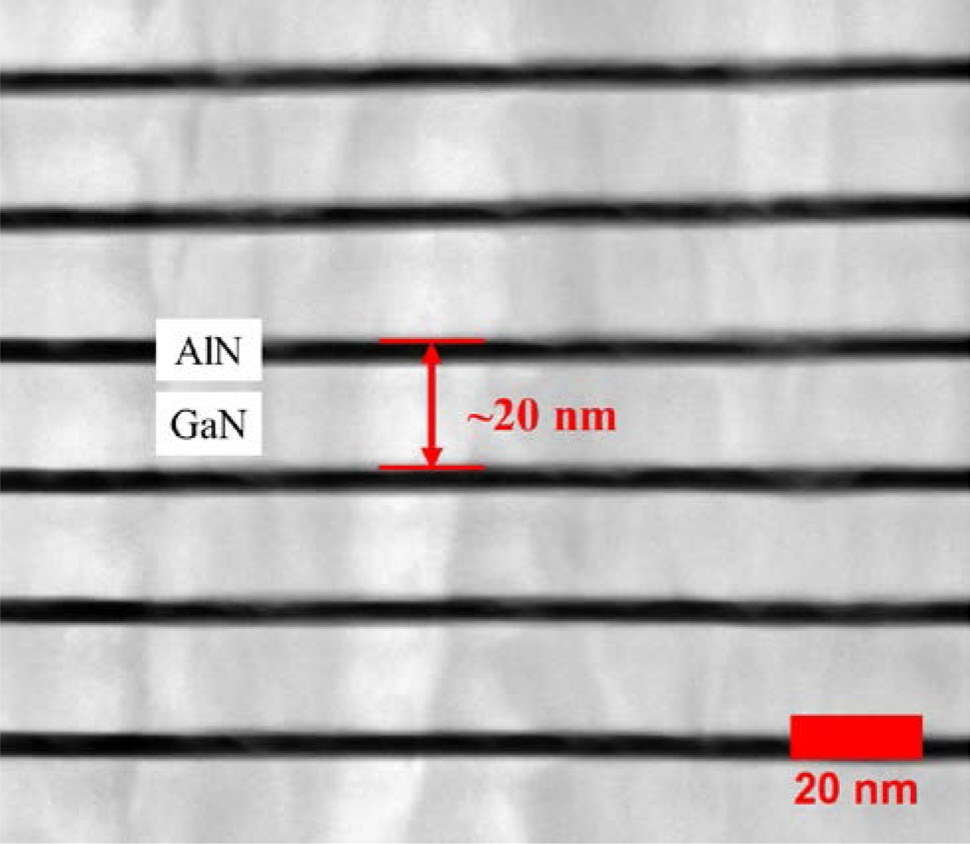
TEM cross sectional images of SSPM-L AlN/GaN.

The HR-XRD phase analysis for 2θ-ω scans of *a*-GaN epitaxial layers with different numbers of SSPM-L AlN/GaN pairs is shown in Fig. 3. The two dominant peaks observed at ~ 52.7° and 57.6° for all the samples corresponded to the diffractions of the *r*-plane sapphire substrate [2–204] and *a-*GaN [11–20], respectively. The clearly observable Pandellosung fringes resulted from the interface formed using different numbers of SSPM-L AlN/GaN pairs^30^. This result showed the abrupt structure and good crystalline quality of the SSPM-L AlN/GaN grown on the *a*-GaN epitaxial layer. The results of simulations performed using Rigaku Global Fit software for the satellite peaks showed that each SSPM-L period was 20 nm thick, which was confirmed by the transmission electron microscopy (TEM) images shown in Fig. 4.

**Figure 5:**
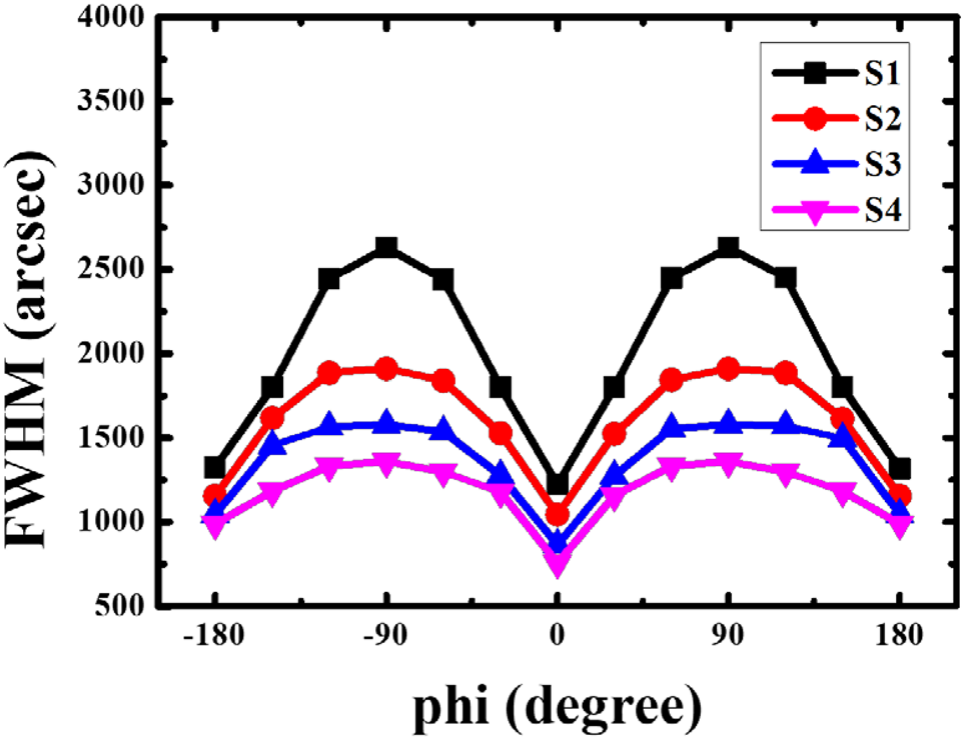
XRC FWHM values from on-axis (11–20) ω-scan as a function of *phi* angle for *a*-GaN grown using 0, 40, 80 and 120 pairs of SSPM-L AlN/GaN.

The crystal quality was further assessed by characterizing the anisotropic properties of the grown *a*-GaN film. Figure 5 shows the FWHM of the ω-scans as a function of the *phi* angles. The XRC ω-scan was performed at *phi* angle intervals of 30°, where the X-ray beam was placed parallel to the *c*- and *m*-axes along the (11–20) plane at 0° and 90° *phi* angles, respectively. The FWHM plot exhibits the same trend as the nonpolar XRC measurements, that is, the anisotropy of nonpolar GaN results in an “M”-shaped plot with respect to the *phi* angle^10,15,31^. The lowest FWHMs are observed at a *phi* angle of 0°, corresponding to 1224, 1044, 864 and 756 arcsec for samples S1, S2, S3 and S4, respectively. The FWHM values broaden with the *phi* angle rotation, and the highest values of 2628, 1911, 1581 and 1360 arcsec are obtained at a *phi* angle of 90° for samples S1, S2, S3 and S4, respectively. The reduction in the FWHM as the number of pairs increases shows that SSPM-L AlN/GaN reduced the defect density in the grown *a*-GaN film. Moram et al. have reported that FWHM broadening can significantly affect the surface morphology of a grown GaN layer, which is in agreement with the FESEM micrographs shown above^32^. Increasing the number of SSPM-L AlN/GaN pairs further improved the FWHM ratios from 90° to a minimum of 0°, namely, to 2.14, 1.86, 1.83 and 1.80 for samples S1, S2, S3 and S4, respectively. This result indicates that isotropy enhancement reduced the distortion of the wurtzite crystal structure caused by the enormous twist and tilt mosaicity along the *c*- and *m*-directions^33^. Hence, it is deduced that the FWHM decrements reflected a reduction in the number of surface voids and the roughness as the number of SSPM-L AlN/GaN pairs increased.

**Figure 6:**
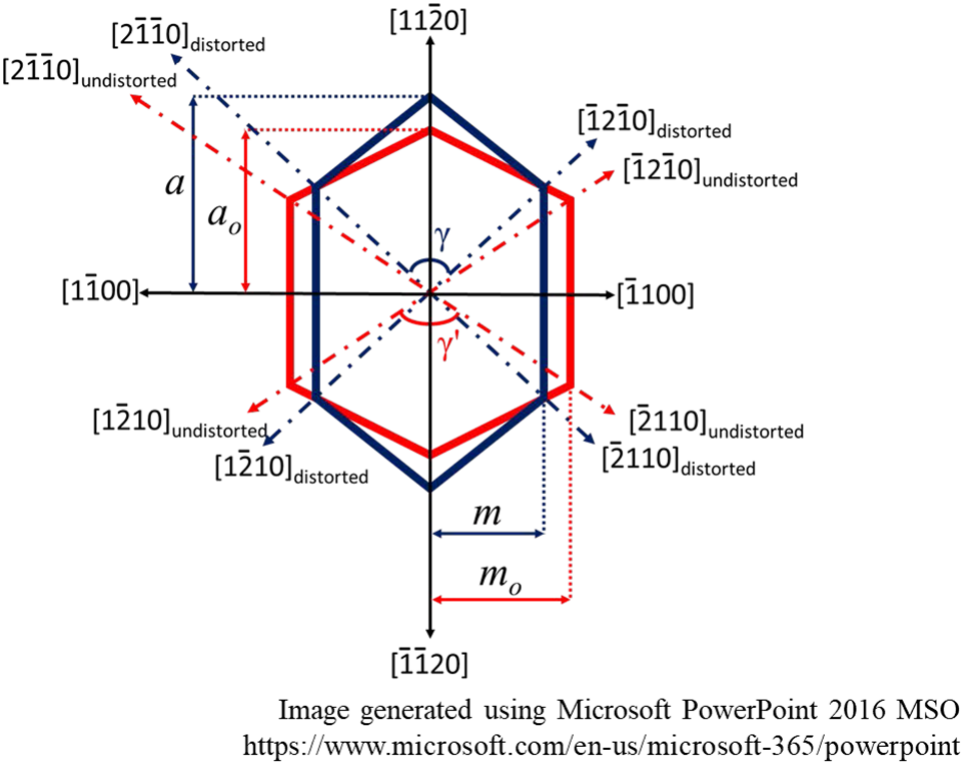
Schematic of perfect (red line) and distorted (dark blue line) wurtzite crystal structure.

Note that the anisotropy of nonpolar GaN induces orthorhombic distortion in the grown *a*-GaN^11,15,34^. The asymmetric crystal arrangement consequently distorts the wurtzite structure^18^. In Fig. 6, the red line represents a perfect crystal arrangement for the wurtzite structure, and the blue line represents the distorted crystal structure resulting from the *a*-GaN film grown in this study. The 2θ values for the (2–1–10), (–12–10) and (11–20) planes were measured to determine the interplanar distance (d_*hkil*_) using Bragg’s law (d_hkl_ = λ/2 sin θ_hkl_)^35^. The 2θ values for the (2–1–10), (–12–10) and (11–20) planes of all the samples are listed in Table 1. The 2θ values for the (2–1–10) and (–12–10) planes are similar but differ significantly from that of the (11–20) plane. These results suggest that large lattice stretching within the [2–1–10] and [–12–10] directions leads to hexagonal symmetry angle distortion and persistent orthorhombic distortion of the grown GaN. Increasing the number of SSPM-L AlN/GaN pairs results in a linear decrease in the 2θ values for the (2–1–10) and (–12–10) planes to nearly that of undistorted GaN, reflecting a reduction in the distortion of the crystal structure. Hence, the distorted angle (γ’) depends strongly on the offset of the basis angle (δ), which corresponds to the difference between γ and γ’.

**Table 1:** Value of 2θ from phase analysis along (2–1–10), (–12–10) and (11–20) planes.

Number of pairs	2θ (°)		
	(2–1–10)	(–12–10)	(11–20)
0	58.107	58.106	57.767
40	57.986	57.984	57.656
80	57.957	57.956	57.637
120	57.929	57.929	57.619

**Figure 7:**
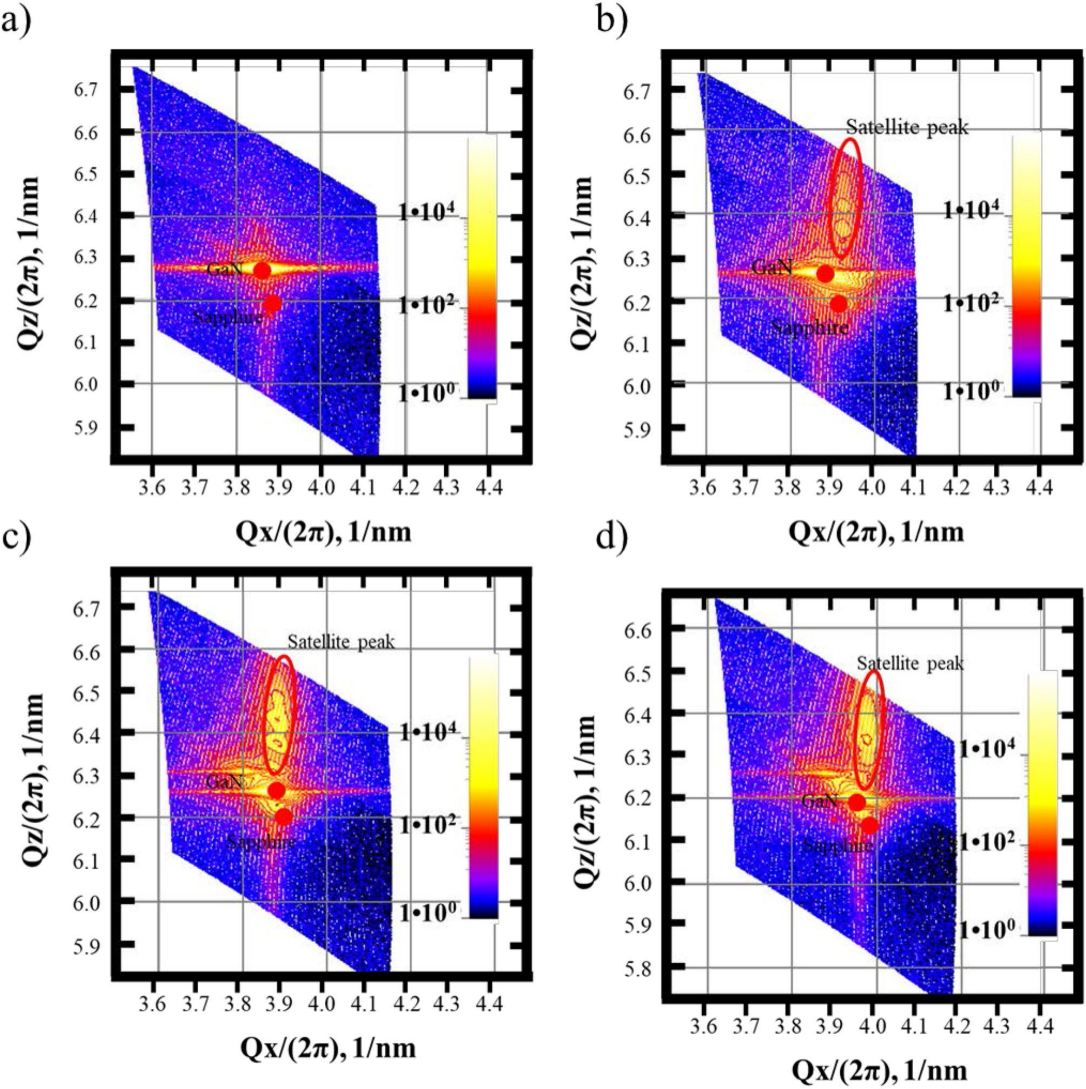
HR-XRD RSM scan along (11–22) for samples (**a**) S1, (**b**) S2, (**c**) S3 and (**d**) S4.

Figure 7a–d shows the HRXRD RSM along the [11–22] direction that was measured for all the samples to investigate the lattice size and strain state of the grown *a*-GaN film. The RSM measurement was performed along the (11–22) plane, because the interplanar spacing coordinates of *q*_*x*_ and *q*_*z*_ are proportional to 1/*c* and 1/*a*. This measurement was used to determine the lattice size in both the *c* and *a* directions. Figure 7a shows a single dominant peak for sample S1, which is ascribed to GaN, and a weak sapphire peak. The RSM images for samples S2, S3 and S4 shown in Fig. 7b–d consist of satellite peaks of SSPM-L AlN/GaN and a sapphire peak. The *q*_*x*_ components of the *a*-plane GaN peaks for samples S1, S2, S3 and S4 are 3.863, 3.864, 3.957 and 3.862 (2π/nm), respectively. The corresponding *q*_*z*_ components are 6.261, 6.259, 6.201 and 6.273 (2π/nm). The differences in both the *q*_*x*_ and *q*_*z*_ components indicate that the samples have different lattice sizes in both the *a* and *c* directions.

The least-squares method developed by Laskar et al*.* was used to perform a highly accurate calculation of the distortion angle, which plays an important role in lattice determination, especially in the *m*-direction^11^. As the *a*-GaN thin films grown in this study exhibit orthorhombic distortion (see Table 1), the lattice parameters for the distorted *a*-GaN structure are determined using Eq. (1):

1 $$\frac{1}{{d}_{hkl}^{2}}=\left[\frac{4}{3}\frac{({h}^{2}+{k}^{2}+hk)}{{a}^{2}}\right]-\left[\frac{4}{3\sqrt{3}}\frac{\left(2{h}^{2}+2{k}^{2}+5hk\right)}{{a}^{2}}\delta \right]+\frac{{l}^{2}}{{c}^{2}}$$

where *h, k* and *l* are the Miller indices; *a* and *c* are the lattice constants; and δ is the offset in the basis angle, which was used to determine the distortion angle and the lattice parameter in the *m-*direction. The strains in the (11–20), (0001) and (1–100) directions are determined using the equations given below^34^:

2 $${\varepsilon }_{xx}=\frac{a-{a}_{0}}{{a}_{0}}$$

3 $${\varepsilon }_{zz}=\frac{c-{c}_{0}}{{c}_{0}}$$

4 $${\varepsilon }_{yy}=\frac{m-{m}_{0}}{{m}_{0}}$$

where *a*_*o*_, *c*_*o*_ and *m*_*o*_ represent the lattice constants of the fully relaxed GaN structure, as shown in Table 2; ε_xx_ is the nominal out-of-plane strain in the [11–20] direction for *a*-GaN; and ε_zz_ and ε_yy_ are both in-plane strains along the *c*- and *m*-directions, respectively. As the stress along the growth direction is naturally zero^11,34^, the stress along the *c*- and *m*-directions can be measured using the following equations:

**Table 2:** Lattice constants and elastic stiffness coefficients for perfect hexagonal GaN.

Lattice parameters			Elastic stiffness constant			
*a*_*0*_ (Å)	*c*_*0*_ (Å)	*m*_*0*_ (Å)	*c*_11_ (Gpa)	*c*_12_ (Gpa)	*c*_13_ (Gpa)	*c*_33_ (Gpa)
3.1893	5.1851	2.762	390	145	106	398

5 $${\sigma }_{zz}={C}_{13}{\varepsilon }_{xx}+{C}_{13}{\varepsilon }_{yy}+{C}_{33}{\varepsilon }_{zz}$$

6 $${\sigma }_{yy}={C}_{12}{\varepsilon }_{xx}+{C}_{11}{\varepsilon }_{yy}+{C}_{13}{\varepsilon }_{zz}$$

where σ_zz_ and σ_yy_ are the stresses along the *c*- and *m*-directions, respectively; and *Cij* are the elastic stiffness coefficients presented in Table 2. All the data related to lattice parameters, strain and stress states calculated from the RSM scan are tabulated in Table 3.

**Table 3:** Lattice parameters for grown GaN obtained from RSM scan along (11–22) plane.

Number of pairs	*a*(Å)	*c*(Å)	*m*(Å)	γ (°)	ε_xx_(%)	ε_zz_(%)	ε_yy_(%)	σ_zz_ (Gpa)	σ_yy_ (Gpa)
0	3.195	5.175	2.764	119.782	+ 0.182	− 0.181	+ 0.072	+ 0.287	− 0.156
40	3.194	5.177	2.763	119.779	+ 0.164	− 0.155	+ 0.053	+ 0.222	− 0.155
80	3.191	5.178	2.761	119.778	+ 0.062	− 0.138	− 0.049	− 0.278	− 0.646
120	3.188	5.179	2.758	119.777	− 0.040	− 0.122	− 0.152	− 0.776	− 1.137

+ tensile; − compressive.

The grown *a*-GaN film has different lattice constants *a, c* and *m* for different numbers of SSPM-L AlN/GaN pairs, which are in good agreement with the aforementioned RSM measurements. This result shows that the number of SSPM-L AlN/GaN pairs can be varied to control both the lattice size and the degree of the distortion of nonpolar *a*-GaN. Table 3 shows the out-of-plane strain along the growth direction [11–20]. The out-of-plane strain along the growth direction [11–20] tends to decrease as the number of SSPM-L AlN/GaN pairs increases. The out-of-plane strain along the [11–20] direction is in a tensile state and changes to a compressive state once the number of SSPM-L AlN/GaN pairs reaches 120, as shown in Table 3. These results are in good agreement with a report by Yiqiang et al. that increasing the number of AlN/GaN pairs changes the strain state of the GaN surface from tensile to compressive^36^.

**Figure 8:**
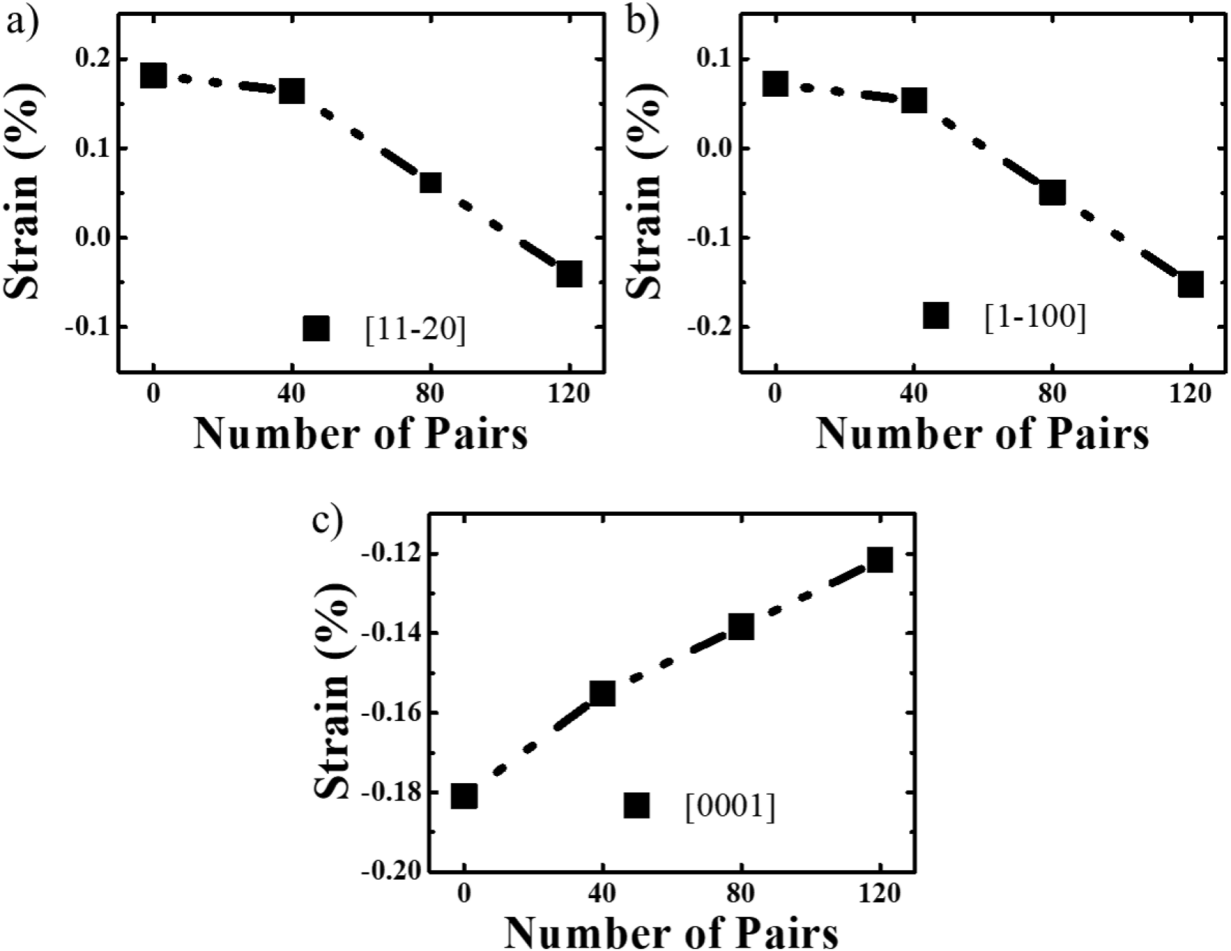
Strain state for GaN grown with different numbers of SSPM-L AlN/GaN pairs along (**a**) [11–20], (**b**) [1–100] and (**c**) [0001] directions.

Figure 8a–c shows the strain state of the *a*-GaN samples grown with different numbers of SSPM-L AlN/GaN pairs along the [11–20], [1–100] and [0001] directions. A similar trend in the strain along [11–20] and [1–100] can be clearly observed: the strain decreases as the number of pairs increases and is transformed from a compressive to tensile state. Figure 8a shows that the strain in the [11–20] direction decreases from 0.17% to 0.05% as the number of SSPM-L AlN/GaN pairs increases, whereas the strain in the [1–100] direction decreases from 0.07% to − 0.15%, as shown in Fig. 8b. Figure 8c shows the compressive strain changes from − 0.18 to − 0.12%. A similar trend in the strain is observed for the hexagonal wurtzite crystal structure along the [11–20] and [1–100] directions, whereas an opposite trend is observed along [0001], because the angles from [11–20] are much closer to [1–100] and completely opposite to the [0001] direction. These results show that the presence of SSPM-L AlN/GaN induces a compressive strain along the [11–20] and [1–100] directions and a tensile strain along the [0001] direction within the epi layer. Figure 8a–c shows that the strain state is highly sensitive to the number of SSPM-L AlN/GaN pairs along the aforementioned directions. This result could be attributed to the difference between the lattice sizes of AlN and GaN in SSPM-L AlN/GaN, which creates a large compressive strain in the GaN epilayer along the nominal growth direction. Therefore, the compressive strain along the [0001] direction increases with the number of pairs in the SSPM-L AlN/GaN structure to counter the tensile strain induced by the difference between the lattices of the *r*-sapphire substrate and the initial layer of grown *a*-GaN. Consequently, a state of near-relaxation strain occurs at the top of the *a*-GaN epitaxial growth. Hence, the grown *a*-GaN exhibits a near-relaxation strain along the [11–20] and [1–100] directions, which increases the strain along the [0001] direction.

**Figure 9:**
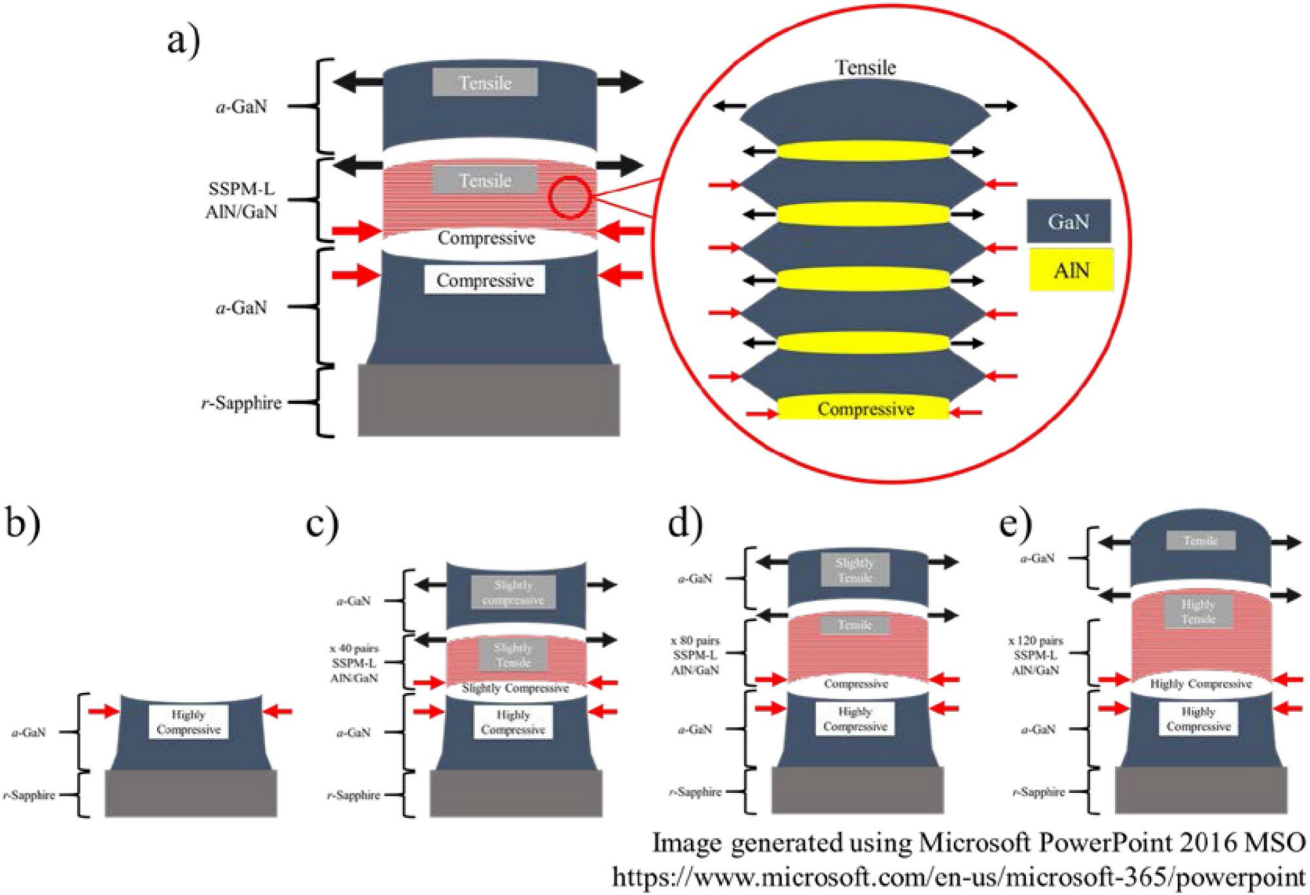
(**a**) Schematic of *a*-GaN growth on *r*-plane sapphire using SSPM-L AlN/GaN and schematized strain growth in samples (**b**) S1, (**c**) S2, (**d**) S3 and (**e**) S4.

Figure 9a is a schematic representation of *a*-GaN growth in the presence of SSPM-L AlN/GaN, in which the strain growth mechanism is elucidated in depth. The direct growth of *a*-GaN on the *r*-sapphire substrate induces a compressive strain in the thin film, particularly in the [0001] direction, as shown in Fig. 9b. This result can mainly be attributed to the large in-plane lattice constants in both the [0001] and [1–100] directions of the sapphire substrate^18^. SSPM-L AlN/GaN induces a large compressive strain in the AlN layer at the bottom of the SSPM-L AlN/GaN stack because of the lattice mismatch between AlN and GaN. The atomic arrangement of the AlN layer within SSPM-L stretches to adjust to the large lattice in the GaN layer in SSPM-L. However, the atomic arrangement of the GaN layer in SSPM-L contracts to adjust to the small lattice of the AlN layer in SSPM-L. The stretching and contraction of the atomic arrangement in SSPM-L AlN/GaN induces a tensile strain state in the topmost GaN layer, resulting from the increase in the tensile strain for consequent *a*-GaN growth. Sample S2 undergoes a slight compressive strain, because the tensile strain for 40 pairs of SSPM-L AlN/GaN is insufficient to completely transform the compressive strain state to the tensile strain state, as shown in Fig. 9c. Increasing the number of SSPM-L AlN/GaN pairs induces a tensile state in the topmost GaN layer, as shown in Fig. 9d and e, which is in accordance with the calculated strain based on the RSM measurement.

**Figure 10:**
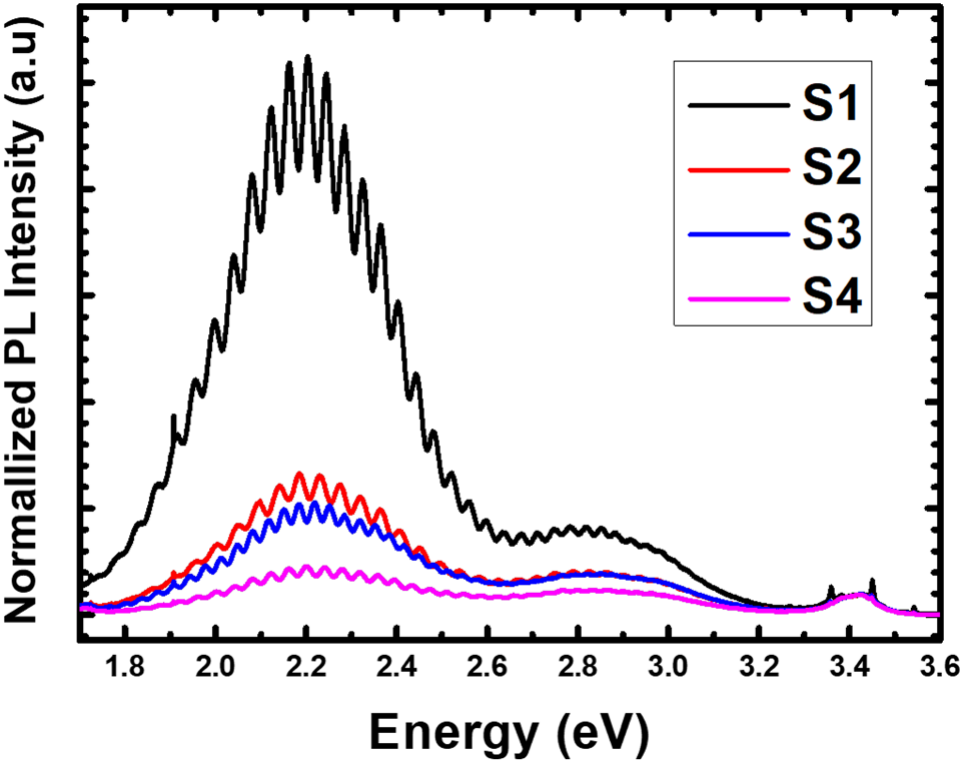
RT-PL spectra for samples S1, S2, S3 and S4.

Photoluminescence (RT-PL) measurements were used to investigate the effect of different numbers of SSPM-L AlN/GaN pairs on the optical properties at room temperature. Figure 10 shows the RT-PL spectra for *a*-GaN grown on different numbers of SSPM-L AlN/GaN pairs. The RT-PL spectra were normalized using the near-band-edge emission (NBE) centred at 3.4-eV. Yellow luminescence (YL) and blue luminescence (BL) bands centred at 2.2 eV and 2.9 eV, respectively, can be clearly discerned for all the samples. Sample S1 exhibits the highest relative intensity for both the YL and BL bands. However, the relative intensities for the YL and BL bands decrease as the number of SSPM-L AlN/GaN pairs increase. Sample S4 exhibits the least intense YL and BL bands, showing that increasing the number of SSPM-L AlN/GaN pairs effectively improves the PL properties of the grown GaN. Other research groups have attributed the YL and BL bands to the formation of gallium particles, nitrogen vacancies and deep level impurities^37–40^. It is safe to presume that the rough surface of the grown *a*-GaN is highly correlated with the occurrence of YL and BL bands, where the FESEM and HRXRD shows improved results with the enhanced PL properties as the number of SSPM-L AlN/GaN pairs increase. The obtained PL spectra are in good agreement with the RSM measurements, that is, the sample without SSPM-L AlN/GaN exhibits the highest on-axis strain as well as the highest YL and BL band intensities. Furthermore, increasing the number of SSPM-L AlN/GaN pairs caused the YL and BL band intensities to weaken, which promoted an on-axis near-relaxation strain state. Dislocations and defects tend to create a local strain field within a crystal structure to produce a one-dimensional electronic potential^38^. Consequently, the binding excitations of the PL reflection increase in intensity. The implantation of SSPM-L AlN/GaN promoted the near-relaxation strain state within the crystal structure, which reduced defects and weakened the YL and BL intensities. Several reports on -III-nitride have shown a correlation between the YL and BL bands with carbon impurities^37–40^. As low pressure and low V/III ratio conditions increase the growth window of nonpolar GaN^41–44^, significant unintended carbon doping results from the decomposition of TMG and/or TMA during the growth process. This result is strongly correlated with the on-axis XRC measurements, where the highest FWHM is obtained for *a*-GaN growth without SSPM-L AlN/GaN.

**Figure 11:**
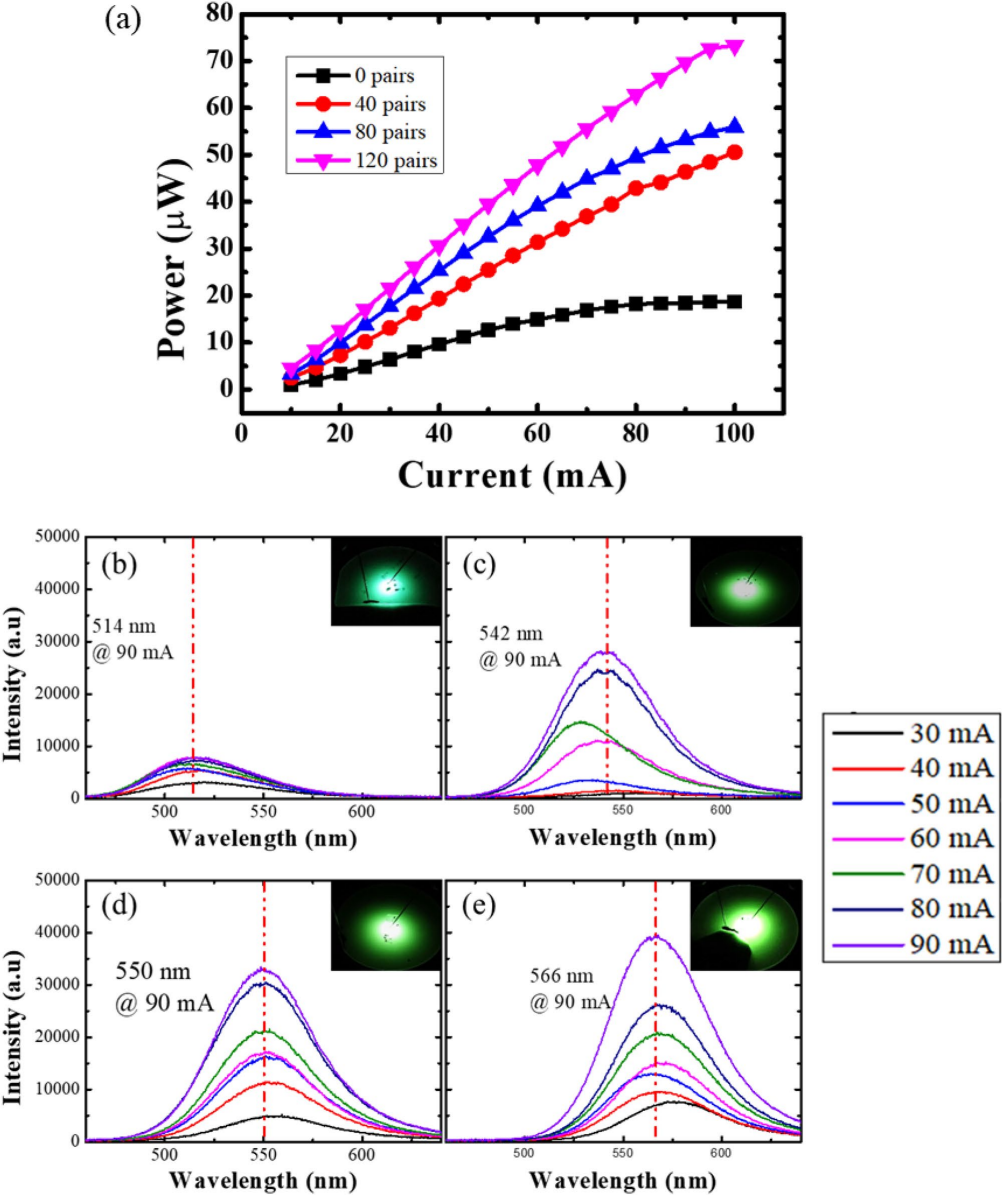
InGaN/GaN LEDs grown on *a*-GaN with different numbers of SSPM-L AlN/GaN pairs: (**a**) light output power measured for substrates with injection currents from 10 to 100 mA and EL spectra measured at injection currents of 20 mA–90 mA for (**b**) 0, (**c**) 40, (**d**) 80 and (**e**) 120 pairs of SSPM-L AlN/GaN.

Finally, the electrical properties of the *a*-GaN LEDs grown with different numbers of SSPM-L AlN/GaN pairs were studied to determine whether the enhancement in the microstructural quality of *a*-GaN makes the thin film viable for use in optoelectronic devices. A total of 5 pairs of InGaN/GaN were grown on the *a*-GaN structure. Then, the I-V characteristics of the chip-on-wafer configuration were determined. The output power-current characteristics are shown in Fig. 11a. The output power of the LEDs grown on S1 increase linearly and begin to saturate at 70 mA. However, the LEDs grown on S2, S3 and S4 show a linear increase in the output power without saturating. The LEDs with 120 pairs of SSPM-L AlN/GaN exhibit the highest output power up to 70 μW at 100 mA compared to 10 μW for the device without SSPM-L AlN/GaN. The *a*-GaN crystal structure has a rough surface morphology and poor crystallinity, such that there is a high tendency for charge carriers to become trapped in the energy levels close to either the conduction or valence bands^45,46^. The resulting significant reduction in the number of hole-electron recombinations within the energy band leads to defect level recombination, thereby increasing phonon generation. This mechanism is consistent with the PL spectra presented in Fig. 10. Consequently, increasing the phonon concentration within the active region leads to additional heat that can induce tensile strain in the InGaN/GaN layer^47,48^. Therefore, increasing the number of SSPM-L AlN/GaN pairs induces a near-relaxation strain in the crystal structure, increasing radiative recombination. These results correlate well with the FESEM and HRXRD results that show an enhanced surface structure and crystalline quality, which significantly increases the emission output power. Figure 11b–e shows the EL emission spectrum of InGaN/GaN LEDs grown on an *a*-GaN film for different injection currents ranging from 30 to 90 mA. Similar trends in the FWHM and the wavelength shift with increasing injection current are observed for all the LEDs. The FWHM starts to narrow and exhibits a slight blueshift in the wavelength emission. The blueshift exhibits the same trend reported by Yon et al. for orange *a*-plane LEDs, where increasing the current resulted in band filling, such that the InGaN/GaN active region saturated to a lower energy. The slight broadening of the FWHM with increasing current could be attributed to space separation within the In/GaN layer that induces compositional fluctuations within the InGaN alloys^49^. The measured FWHM peaks start to narrow as the number of SSPM-L AlN/GaN pairs increases. The *a*-GaN film with 120 pairs of SSPM-L AlN/GaN exhibits the narrowest FWHM, suggesting that the LED structure grown on the *a*-GaN with 120 pairs of AlN/GaN facilitates MQW growth with a higher interfacial abruptness because of lower surface roughness and fewer v-pit defects. The subsequent increase in the electron–hole recombination rate increases the output power. The previously presented results for the crystalline analysis and surface properties show that increasing the number of SSPM-L AlN/GaN pairs contributes significantly to increasing the crystal quality and the device performance. Note that the emission peak wavelength starts to shift from 514 to 566 nm as the number of SSPM-L AlN/GaN pairs increase. There is a redshift in the emission wavelength with the increasing number of SSPM-L AlN/GaN pairs: thus, we consider that the most favourable indium (In) composition probably corresponds to the structure with 120 pairs of SSPM-L AlN/GaN, which exhibits the lowest tensile strain, as shown in Fig. 8. It is challenging to incorporate In in growing GaN-based LEDs, because excessive TMI flow during the growth process induces a compressive strain state in the InGaN/GaN layer^50,51^. Our results suggest that growing an *a*-GaN layer with a large number of SSPM-L AlN/GaN pairs would provide a tensile strain state for In incorporation that facilitates a near-relaxation strain state within the epi layer. The smooth surface and high crystalline quality for a-GaN with 120 pairs of SSPM-L AlN/GaN shown by the FESEM and XRC results facilitate In incorporation during the growth process^52^.

## Conclusions

In conclusion, we successfully grew an *a-*plane GaN film using 0, 40, 80 and 120 pairs of SSPM-L AlN/GaN. Increasing the number of SSPM-L AlN/GaN pairs was demonstrated to effectively reduce the surface roughness and enhance the surface structure and crystalline properties. Moreover, SSPM-L AlN/GaN successfully induced a near-relaxation strain state in both the [0001] and [11–20] directions of the grown (11–20) *a*-GaN film. We also grew InGaN/GaN LEDs on the grown GaN, wherein the *a-*plane InGaN/GaN LED with 120 pairs of SSPM-L AlN exhibited the highest output power (up to 70 μW at 100 mA), as well as highest In incorporation. Although the grown InGaN/GaN LED has a lower output power than conventional *c*-plane LEDs, and this study represents a pioneering step towards further improvement of *a*-plane light emitting devices.
