# Author Correction: Impact of sandwiched strain periodic multilayer AlN/GaN on strain and crystalline quality of *a*-plane GaN

**DOI:** 10.1038/s41598-021-96016-0

**Published:** 2021-08-11

**Authors:** Anas Kamarundzaman, Ahmad Shuhaimi Abu Bakar, Adreen Azman, Al‑Zuhairi Omar, Noor Azrina Talik, Azzuliani Supangat, Wan Haliza Abd Majid

**Affiliations:** 1 Low Dimensional Materials Research Centre, Department of Physics, Faculty of Science, University of Malaya, 50603 Kuala Lumpur, Malaysia; 2 Nanotechnology Research Centre, Department of Physics, Faculty of Science and Mathematics, Universiti Pendidikan Sultan Idris, 35900 Tanjong Malim, Perak Malaysia

Correction to: *Scientific Reports* https://doi.org/10.1038/s41598-021-89201-8, published online: 06 May 2021

The original version of this Article contained an error in Affiliation 2, which was incorrectly given as ‘Nanotechnology Research Centre, Department of Physics, Faculty of Science, University Pendidikan Sultan Idris, 35900, Tanjung Malim, Malaysia’. The correct affiliation is listed below:

Nanotechnology Research Centre, Department of Physics, Faculty of Science and Mathematics, Universiti Pendidikan Sultan Idris, 35900 Tanjong Malim, Perak, Malaysia

The original Article has been corrected.

